# Decline in independence after three years and its association with dietary patterns and IADL-related factors in community-dwelling older people: an analysis by age stage and sex

**DOI:** 10.1186/s12877-021-02332-5

**Published:** 2021-06-26

**Authors:** Sayuri Kodama, Tanji Hoshi, Sugako Kurimori

**Affiliations:** 1 Department of Food and Nutrition Science, Sagami Women’s Junior College, 2-1-1 Bunkyo, Minami-ku, Sagamihara-shi, Kanagawa-ken 252–0383 Japan; 2 Tokyo Metropolitan University, 1-1 Minami-Osawa, Hachioji-shi, Tokyo, 192-0397 Japan; 3 Department of Nursing, Seitoku University, 550 Iwase, Matsudo-shi, Chiba-ken, 271-8555 Japan

**Keywords:** Independence, Dietary pattern, Instrumental activities of daily living (IADL), Cohort study

## Abstract

**Background:**

Few studies have shown age stage and sex differences in the association among dietary patterns and various health factors related to disability in older people. This study aimed to reveal the differences of characteristics, including several dietary patterns, associated with a decline in independence over 3 years in community-dwelling independent older people. Specifically, we examined data by age stage, for people between 65 and 75 years (earlier-stage) and people aged 75 years or above (later-stage), and sex.

**Methods:**

We conducted a nationwide longitudinal study of 25 Japanese prefectures from 2013 to 2016; 2250 participants’ complete data (1294 men and 956 women) were analyzed. Independence was evaluated based on instrumental activities of daily living (IADL) scores (maximum = 12). Dietary patterns were derived from a principal component analysis of the seven food groups. Baseline IADL-related factors linked to independence 3 years later were selected. Multiple logistic regression analysis for having low independence—without a full score of IADL 3 years after baseline—was conducted, adjusted for baseline IADL scores. Finally, to compare differences among age stage and sex groups, we used Multiple-Group Path Analysis.

**Results:**

Participants with a full IADL score 3 years later were classified as high independence (69.6%), and those without the full score were classified as low independence (30.4%). Only the later-stage older peoples’ proportion of low independence 3 years later was significantly higher than those at baseline. A high meat frequency pattern was associated with a significantly higher risk of decline in independence 3 years later in later-stage older women. The earlier-stage older people showed that 18.5 ≤ BMI < 25 was associated with a lower risk, referring to BMI < 18.5. In the later-stage, exercising three or more times a week with enjoyment and fulfillment was associated with a significantly lower risk.

**Conclusions:**

The IADL disability in older people aged 75 and over showed a rapid change. The different characteristics associated with the risk of decline in independence among age stage and sex were revealed. Targeting age stage and sex separately for community-based comprehensive supportive strategies would be necessary for a long life globally.

## Background

Although the Japanese overall life expectancy is the longest globally, the difference between healthy life expectancy and overall life expectancy was 8.84 years for men and 12.35 years for women in 2016 [1]. Older people are generally defined as those aged 65 years or older. The risk of requiring nursing care increases at 75 years or older (later-stage older people). It has been reported that those aged ≥75 years have multiple diseases that precede disability, such as cerebrovascular disorder, cancer, arthropathy, and fracture [2]. In Japan, the Medical Care System for later-stage older people is separate from the National Health Insurance for those aged < 75. Later-stage older people have a duty of 10% copayment, and those aged 70 to 74 years have a 20% copayment, except for those with income comparable to the current workforce (30% copayment) [3]. Much more effort is needed to reduce medical costs for older people.

In 2025, the baby boomer generation in Japan will be 75 years or older, and the proportion of later-stage older people will increase. What is required for this super-aged society is “community-oriented medical care,” emphasizing quality of life for older people [4]. Creating a community-based system that can provide integrated living support, medical and health services, and long-term care prevention, especially for later-stage older people, is an urgent issue [5]. Nonetheless, effectively integrated support methods, understanding the effectiveness and priority of targeting support by age stage and sex separately in older people, have not yet been fully established. The supportive strategies for older people’s disability prevention, as it stands now, have been primarily targeted at older people of all ages, without considering the needs of their age groups, separately [6].

Diet and nutrition are highly associated with the risk of cognitive decline and dementia in older people. Dietary patterns, which are examined by combinations of foods, have many advantages over individual nutrients or foods [7]. A Mediterranean diet, dietary approaches to stop hypertension (DASH) diet [8], and other healthy dietary patterns derived both a priori (e.g., Healthy Eating Index) [9] and a posteriori (e.g., principal component analysis [PCA], cluster analysis) [10] were shown to be associated with less cognitive decline and/or a reduced risk of dementia. A Japanese-type dietary pattern with low consumption of animal protein, such as beef and pork, and high rice consumption, miso soup, and fish was also related to a healthy life expectancy with independence [11].

There might be age stage and sex differences in the relationship between dietary patterns and older people’s health. A Canadian cohort study conducted on older people aged 67 to 84 showed that adhering to unhealthy Western dietary patterns was related to poorer baseline cognitive function in men but not in women [12]. Another Canadian study conducted for those aged 85 years or older (super-seniors: SS) reported that “Western dietary pattern,” characterized by high-fat food including red meat, was associated with increased odds of being an SS. However, “nutrient-rich” dietary patterns did not remain significant in an adjusted model [13].

Additionally, health status, including older people’s diet quality, might be determined by the complex influence of various factors [4, 14–17]. For example, social participation is strongly associated with older people, resulting in reduced prevention [14]. Hoshi [15] clarified that there is an indirect relationship between socioeconomic status (SES) and healthy life expectancy mediated by the effects of environmental quality, mental, physical, and social health. Moreover, dietary patterns, which have a strong impact on older people’s independence, might also be determined by multiple factors [16, 17]. The authors in this study revealed that the associations between dietary diversity patterns and subjective health were mediated by mental and emotional well-being factors among older Japanese farm villages [16]. It has also been reported that higher education levels and favorable lifestyle are predictors of increased diet quality [17]. Therefore, considering other factors related to older people’s individual and social factors, such as physical activity, subjective health, cognitive function, and community involvement, it is crucial to investigate effective community-based integrated support focusing on dietary patterns. However, few studies have shown the comprehensive association of dietary patterns, especially for those aged 75 years or older, separately. Furthermore, it was indicated that it might be useful to investigate the impact of several dietary patterns simultaneously to elucidate relationships with health outcomes [7].

Therefore, we conducted a nationwide longitudinal survey of community-dwelling older people in Japan to assess respondents’ instrumental activities of daily living (IADL) at baseline and 3 years later, and evaluated changes in the degree of their independence. We comprehensively analyzed the association between a decline in independence over 3 years, several dietary patterns, and IADL-related factors in community-dwelling independent older people, as analyzed by the age stage of two groups: people aged 65–75 years (earlier-stage older people) and people aged 75 years or older (later-stage older people) and sex. This study aimed to reveal the differences of characteristics of the factors, including several dietary patterns, associated with a decline in independence over 3 years in community-dwelling independent older people. Specifically, we examined data by age stage, for people between 65 and 75 years (earlier-stage) and people aged 75 years or above (later-stage), and sex. The findings would contribute to providing evidence on the future supportive strategies for preventing the decline in older people’s independence, targeting age stage and sex separately.

## Methods

### Study participants

This longitudinal survey, which was planned and conducted in collaboration with the Foundation of Social Development for Senior Citizens (FSDSC, Tokyo) and Tokyo Metropolitan University, involved 25 prefectures in Japan. In 2013, a baseline survey of 9508 residents, who had taken part in healthy longevity events carried out by the FSDSC, was conducted (response rate: 45.7%). The FSDSC conducts various projects, e.g., The National Health and Welfare Festival “*Nenrin-pic*,” since 1988, and research to promote well-being and healthy longevity support among older people. The FSDSC staff sent a questionnaire to the participants in person or by mail. A follow-up survey using the same questionnaire was conducted 3 years later. It involved 3990 respondents from the baseline survey who consented to cooperate in the second and subsequent surveys (response rate: 92.6%). A previous study described the details of this survey [18].

From the 3693 valid respondents in both surveys, we excluded participants younger than 65 years (*n* = 510) and those who died (*n* = 35). We also excluded missing data (*n* = 866) for age (*n* = 24), the questions regarding IADL index both at baseline (*n* = 198) and 3 years later (*n* = 151), those related to dietary intake frequency (*n* = 341), and IADL-related factors (*n* = 152). The participants’ independence was evaluated using the IADL score [19]. In the current study “disability” was operationally defined as participants with an IADL score (12 full scores) of less than 9, as described in detail below. After excluding 32 individuals, we analyzed the responses of 2250 people (men: 1294, women: 956) who were identified as independent.

### Evaluation of independence and classification of study participants

We used the 13-item Tokyo Metropolitan Institute of Gerontology Index of Competence (TMIG-IC) to assess independence. It evaluates multiple dimensions of IADL, including intellectual and social ADL, among older people [19]. In this study, the TMIG-IC was modified to contain 12 items (Table 1). We converted two questions, “Do you read newspapers?” and “Do you read books or magazines?” into a single item, “Do you read newspapers or books?” The responses of “Yes” and “No” were scored as 1 and 0, respectively, and were summated to obtain each respondent’s IADL score. The higher the IADL score (up to 12 points), the higher an individual’s degree of independence.

**Table 1:** Instrumental ADL: IADL questionnaire

No.	Questions
**Instrumental Self-Maintenance**	
**1**	Can you use public transportation (bus or train) by yourself?
**2**	Are you able to shop for daily necessities by yourself?
**3**	Are you able to prepare meals by yourself?
**4**	Are you able to pay bills?
**5**	Can you handle your own banking?
**Intellectual Activity**	
**6**	Are you able to fill out forms for your pension?
**7**	Do you read newspapers or books?
**8**	Are you interested in news stories or programs dealing with health?
**Social Role**	
**9**	Do you visit the homes of friends?
**10**	Are you sometimes called on for advice?
**11**	Are you able to visit sick friends?
**12**	Do you ever talk to young people yourself?

The answer is either “yes” or “no.” Each answer will be given 1 point for “yes”

Referring to the TMIG index of Competence from Koyano W, et al. [19]

Since no cutoff point has been reported for the 13-item TMIG-IC [19], we referred to Fujiwara’s study, which defined participants with scores above 10 IADL at baseline to be almost independent in daily living [20]. As mentioned above, we converted two questions into a single question and modified the scale to contain 12 items. Therefore, to decide on the cutoff point for disability in this study’s participants, we examined the relationship with the response rate of questions for each IADL evaluation item for the participants with IADL scores of 8, 9, and 10 points (chi-square test). The evaluation items that showed statistically significant relationships were Instrumental Self-Maintenance (5 full scores) and Social Role (4 of full score). The percentage of participants who were able to do all items of the Instrumental Self-Maintenance questions was significantly lower in the participants who had IADL scores of 8 points (33.3%) than those of 9 points (58.7%) and 10 points (72.5%). Besides, no one had a full score on the Social Role questions in the 8-point participants. Their score was significantly less than those with 9 points (7.9%) and 10 points (8.2%). From the results of these tests, those with 8 points were considered to have a low degree of independence. Therefore, in this study, disability was operationally defined as participants with IADL scores lower than 9 with a modified 12-item index. The participants’ IADL scores 3 years after the baseline were calculated. The group was then divided into two groups: the full score formed the high independence group, and the other, without a full score, formed the low independence group.

### Evaluation of dietary intake frequency

Dietary habits were collected using the frequency of consumption of food groups in the cooking units per week. Seven food groups (meat dishes, soy products, eggs/egg dishes, bluefish, dairy products, fruits, and vegetable dishes) were examined. The frequent consumption of bluefish, which is rich in n-3 fatty acids, is related to the maintenance of older people’s cognitive functions.

Scoring was based on an ordinal scale: 5 points for “every day,” 4 points for “5–6 days a week,” 3 points for “3–4 days a week,” 2 points for “1–2 days a week,” and 1 point for “never.” We examined whether these scores were adequate predictors of participants’ independence 3 years after baseline. The association was analyzed using the sum of all scores as the dietary diversity score—the group who had low independence 3 years after baseline was set as the dependent variable. The logistic regression analysis, adjusted for sex and age, showed that the dietary diversity scores were significantly related to the prevention of low independence 3 years after baseline (odds ratio [OR] = 0.947, 95% confidence interval [CI]: 0.926–0.969). Therefore, this study found that a higher score for dietary intake frequency signaled that a respondent was more likely to maintain independence 3 years from baseline.

### Analysis of IADL-related factors

The items related to IADL were selected referring to the “Questionnaire for the Elderly” [21], provided by the Japanese Ministry of Health, Labour, and Welfare, to evaluate later-stage health conditions of older people. The items used in this analysis included (1) health status, (2) psychological and mental health status, (3) weight change, (4) exercise indices related to falls, (5) smoking, and (6) social participation. (1) For the health condition, “no hospitalization over the previous year” (yes, no) was used. (2) Ordinal scale scores for subjective health (1 = unhealthy, 2 = somewhat unhealthy, 3 = somewhat healthy, 4 = excellent) and life satisfaction (1 = not satisfied, 2 = neither nor, 3 = satisfied) were used to identify psychological and mental health conditions. (3) Body mass index (BMI) was calculated using self-reported height and weight and was classified into four categories: BMI < 18.5, 18.5 ≤ BMI < 25, 25 ≤ BMI < 30, and BMI ≥ 30. (4) For exercise frequency, we used the index of “exercise frequency with enjoyment and fulfillment” [18] that we developed for a previous study for the same participants. Besides the high frequency of exercise for older people, enjoyment and fulfillment are strongly related to maintaining independence 3 years from baseline [18]. There were two questions. The first asked, “How much do you exercise or play sports?” We classified each respondent as “exercises three or more times a week (≥3 times/week)” or “exercises two or fewer times a week (≤2 times/week).” Next, we asked the participants about their enjoyment and fulfillment. If they responded that they enjoyed their exercise, we reclassified them as “exercises three times or more per week with enjoyment and fulfillment (≥3 times/week, E&F)” (or not) and “exercises twice or less per week with enjoyment and fulfillment (≤2 times/week, E&F)” (or not). Finally, four categories were created for the exercise index. (4) We also inquired about “no fall fractures” that had occurred over the previous year (or not). (5) We asked the participants to self-report their cigarette smoking status (current, former, never). (6) The respondents’ social participation was tallied through the frequency of their community and volunteer activity engagement (yes, sometimes, or no). We examined the above baseline items to determine if they were related to independence 3 years later, and we used them in the multivariable analysis if significant. BMI categories did not show a significant single relationship, but we used it as an adjusting factor.

### Other variables

We also analyzed variables related to demographic and SES, including age, sex, the household status of living alone (yes, no), ordinal scaled scores for economic satisfaction (1 = not satisfied, 2 = not very satisfied, 3 = moderately satisfied, 4 = satisfied), and salaried employees (yes, no). We used economic satisfaction as an alternative SES variable instead of annual income because older people’s subjective economic status is more closely related to psychological health than annual income [22].

### Analysis

Comparing the proportion of independence between baseline and 3 years later by age stage and sex groups was analyzed by conducting McNemar’s test. The baseline characteristics were compared between the high- and low-independence groups after 3 years using the Mann–Whitney U test for ordinal scaled scores (including dietary intake frequency). Chi-squared tests were used for all the other variables. To identify dietary patterns, we performed PCA using the dietary intake frequency scores for seven types of food groups. PCA is the most common data-driven method used to derive dietary patterns. Its analytic advantage is that it results in a continuous score, maximizing statistical power to examine the relationship between diet and disease [7].

With the IADL score after 3 years as the dependent variable, two-way ANOVA was conducted for all variables to verify whether there was an effect-modification (interaction) due to the baseline age stage or sex. Multivariate logistic regression analysis was conducted to evaluate the associations among decline in independence after 3 years, dietary patterns, and IADL-related factors [23]. The principal component scores, which are uncorrelated with each other, were used to evaluate the dietary patterns. This analysis’s outcome variable had low independence—without a full score of IADL—after 3 years. ORs and 95% CIs were calculated separately for age stage and sex. The multivariate models were adjusted for IADL scores at baseline. In addition, to compare differences among age stage and sex groups, Multiple-Group Path Analysis was conducted for age stage, sex, and their combination, simultaneously [15, 24, 25]. Path Analysis is one of the methods of a causal modeling approach for Structural Equation Modeling (SEM). We examined the direct effects of baseline characteristics on low independence 3 years later. We used the same indicators examined in the multiple logistic regression analysis, adjusting for the effect of each indicator. Standardized estimates (S.E.) that compare the magnitude of the effect between groups were calculated. Then all effects were labeled in order, and a comparison of the label’s pair was generated with a critical ratio (CR). CR presents a ratio associated with differences between two effects. A statistically significant difference was indicated by a CR > ±1.96: *P* < 0.05, CR > ±2.23: *P* < 0.01, and CR > ±2.58: *P* < 0.001.

The statistical analyses were performed using SPSS Statistics 24.0. Multiple-Group Analysis was conducted using Amos statistical software 23.0 for Windows. Statistical significance was set at *P* < 0.05.

## Results

### IADL score of baseline and 3 years later

The study participants were 1294 men (57.5%) and 956 women (42.5%) with a total of 2250 independent older people. The baseline age composition was 1545 (68.7%) earlier-stage older people (men: 863, 55.9%; women: 682, 44.1%) and 705 (31.3%) later-stage older people (men: 431, 61.1%; women: 274, 38.9%). The average age was 72.32 ± 5.22 years (men: 72.56 ± 5.43 years, women: 72.01 ± 4.92 years).

**Fig. 1:**
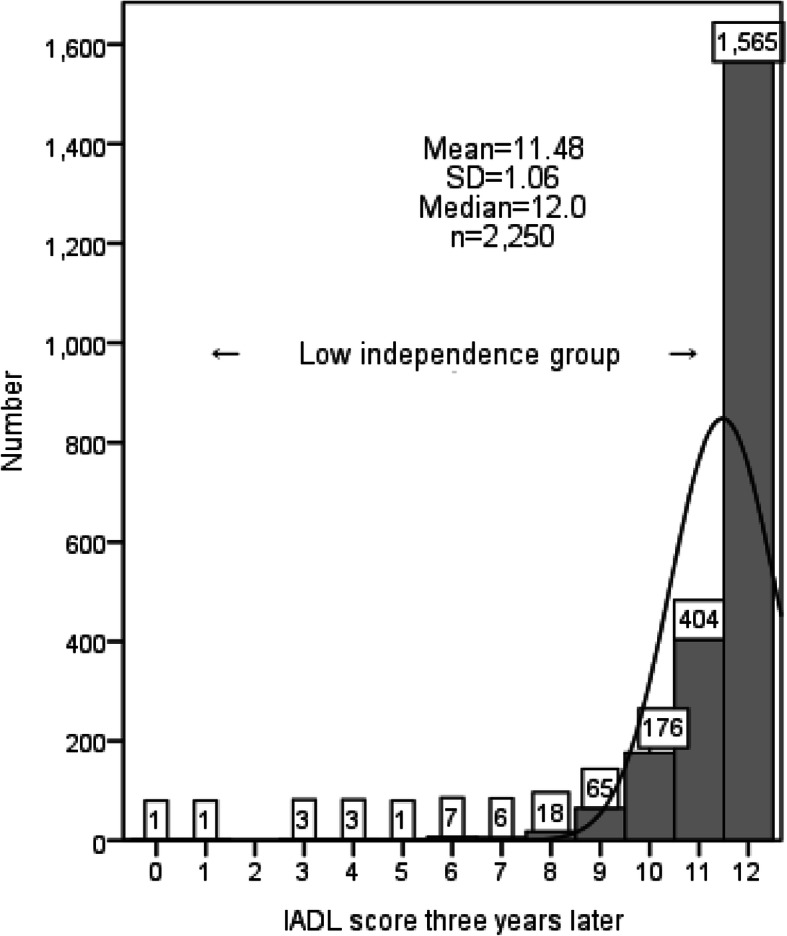
Distribution of the IADL score 3 years later. IADL, Instrumental Activities of Daily Living

The mean IADL score 3 years after baseline was 11.48 ± 1.06, and its median was 12 full scores. The distribution of the IADL score 3 years later is shown in Fig. 1. Participants with a score of 0 were admitted to a medical institution. Participants with a full score of 12 points 3 years after baseline, who were completely independent, were classified as “high independence” (*n* = 1565, 69.6%). The others without a full score of IADL, who were not completely independent, were classified as “low independence” (*n* = 685, 30.4%). The proportion of low independence respondents 3 years after baseline was 37.5% for men, 20.9% for women, 28.3% for earlier-stage older people, and 35.2% for later-stage older people. Of the low independence participants, 67.0% had no change 3 years from baseline, and 33.0% changed to high independence. Whereas 17.2% of the high independence group changed to low independence, and 82.8% maintained their status 3 years later.

**Fig. 2:**
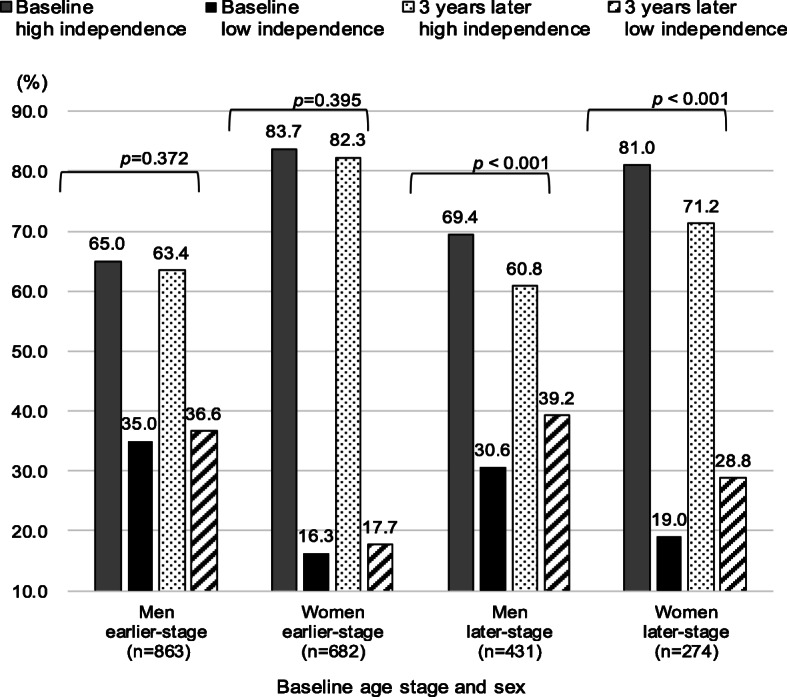
Comparison of proportion of independence between baseline and 3 years later

Comparing the proportion of high and low independence between baseline and 3 years later by age stage and sex in the two groups, only the later-stage older peoples’ results were significantly different (Fig. 2). It was shown that the proportion of low independence 3 years later was higher than those at baseline (later-stage men: baseline 30.6%, 3 years later 39.2%; later-stage women: 19.0, 28.8%).

### Relationship between baseline-related factors and independence 3 years later

Table 2 shows the relationship between the baseline values and independence 3 years later. It compares the high independence groups’ results with the low independence group. The analysis was performed for each age stage. The main item with which only earlier-stage older people showed a significant relationship was smoking. The percentage of those who had never smoked in the high independence group 3 years after baseline was the highest at 77.8%. The proportion of those who smoked and reached low independence was 44.4%, the highest among the low independence group.

**Table 2:** Relationship between baselines characteristics and independence 3 years later

	All participants (*n* = 2250)					Earlier-stage (*n* = 1545)					Later-stage (*n* = 705)				
Baseline characteristics	High independence		Low independence			High independence		Low independence			High independence		Low independence		
	n	%	n	%	*P* value	n	%	n	%	*P* value	n	%	n	%	*P* value
**Sex**	1565	69.6	685	30.4		1108	71.7	437	28.3		457	64.8	248	35.2	
Men	809	62.5	485	37.5	< 0.001	547	63.4	316	36.6	< 0.001	262	60.8	169	39.2	0.003
Women	756	79.1	200	20.9		561	82.3	121	17.7		195	71.2	79	28.8	
**Household status**															
Living alon	213	72.0	83	28.0	0.344	134	76.6	41	23.4	0.154	79	65.3	42	34.7	1.000
Living alon, No	1352	69.2	602	30.8		974	71.1	396	28.9		378	64.7	206	35.3	
**Economic satisfaction**															
Satisfied	414	71.6	164	28.4	0.093	276	76.2	86	23.8	0.046	138	63.9	78	36.1	0.596
Moderate satisfied	873	69.6	382	30.4		611	70.7	253	29.3		262	67.0	129	33.0	
Not very satisfied	225	67.2	110	32.8		173	68.1	81	31.9		52	64.2	29	35.8	
Not satisfied	53	64.6	29	35.4		48	73.8	17	26.2		5	29.4	12	70.6	
**Salaried employee**															
Yes	336	72.9	125	27.1	0.045	258	73.1	95	26.9	0.281	78	72.2	30	27.8	0.049
No	1229	68.7	560	31.3		850	71.3	342	28.7		379	63.5	218	36.5	
**Hospitalization over the past year**															
Yes	160	62.7	95	37.3	0.008	103	63.6	59	36.4	0.011	57	61.3	36	38.7	0.257
No	1405	70.4	590	29.6		1005	72.7	378	27.3		400	65.4	212	34.6	
**Subjective health**															
Excellent	297	76.5	91	23.5	< 0.001	217	78.3	60	21.7	0.005	4	44.4	5	55.6	< 0.001
Somewhat healthy	1185	69.4	523	30.6		826	70.6	344	29.4		14	29.8	33	70.2	
Somewhat unhealthy	67	54.0	57	46.0		53	68.8	24	31.2		359	66.7	179	33.3	
Unhealthy	16	53.3	14	46.7		12	57.1	9	42.9		80	72.1	31	27.9	
**Life satisfaction**															
Satisfied	1316	71.0	538	29.0	0.001	927	73.0	343	27.0	0.017	389	66.6	195	33.4	0.025
Neither nor	209	63.3	121	36.7		151	65.7	79	34.3		58	58.0	42	42.0	
Not satisfied	40	60.6	26	39.4		30	66.7	15	33.3		10	47.6	11	52.4	
**BMI categories**															
BMI < 18.5	58	59.2	40	40.8	0.107	34	64.2	19	35.8	0.240	24	53.3	21	46.7	0.121
18.5 < BMI < 25.0	1227	70.5	514	29.5		871	72.6	328	27.4		357	65.7	186	34.3	
25.0 < BMI < 30.0	262	68.1	123	31.9		195	70.1	83	29.9		67	62.6	40	37.4	
30.0 < BMI	18	69.2	8	30.8		8	53.3	7	46.7		9	90.0	1	10.0	
**Exercise frequency, enjoyment & fulfillment (E&F)**															
3 times < / week, E&F	794	71.5	316	28.5	0.147	564	72.6	213	27.4	0.896	230	69.1	103	30.9	0.039
3 times < / week	104	69.8	45	30.2		67	71.3	27	28.7		37	67.3	18	32.7	
2 times > / week, E&F	406	68.5	187	31.5		287	70.9	118	29.1		119	63.3	69	36.7	
2 times > / week	261	65.6	137	34.4		190	70.6	79	29.4		71	55.0	58	45.0	
**Fall fractures in the past year**															
Yes	155	58.7	109	41.3	< 0.001	99	61.9	61	38.1	0.003	56	53.8	48	46.2	0.008
`No	1410	71.0	576	29.0		1009	72.9	376	27.1		401	66.7	200	33.3	
**Cigarette smoking status**															
Current	51	58.0	37	42.0	< 0.001	40	55.6	32	44.4	< 0.001	11	68.8	5	31.2	0.433
Former	493	63.0	289	37.0		340	63.3	197	36.7		153	62.4	92	37.6	
Never	1021	74.0	359	26.0		728	77.8	208	22.2		293	66.0	151	34.0	
**Community and volunteer activity engagement**															
Yes	877	75.3	288	24.7	< 0.001	608	77.7	174	22.3	< 0.001	269	70.2	114	29.8	< 0.001
Sometimes, No	688	63.4	397	36.6		500	65.5	263	34.5		188	58.4	134	41.6	

Having salaried employment was one of the main items in which only later-stage older people showed a significant relationship. The percentage of salaried employees who were among the high independence group 3 years later was the highest at 72.2%. Contrastingly, the proportion of those without a low independence salary reached 36.5%, the highest among the low independence respondents. The results of the exercise frequency of the later-stage older people showed that the percentage of those who exercised ≥3 times/week with E&F and were identified as having high independence 3 years after baseline was the highest at 69.1%. Conversely, the proportion of those who exercised 2 times ≥ a week without E&F and had low independence was 45.0%, the highest among the low independence respondents.

### Relationship between dietary intake frequency and independence 3 years later

Table 3 shows the relationship between baseline dietary intake frequency and independence 3 years later. It compares the high independence groups’ results with those of the low independence group. Overall, the high independence group was significantly higher in each food group’s intake frequency than the low independence group. However, the percentage of those who ate meat every day and reached low independence 3 years later was high among the low independence group (overall result: 32.7%). Further, it did not differ significantly from those who ate meat 1–2 days a week (32.7%) or did not eat meat (32.8%).

**Table 3:** Relationship between dietary intake frequency and independence 3 years later

	All participants (n = 2250)					Earlier-stage (n = 1545)					Later-stage (n = 705)				
Baseline characteristics	High independence		Low independence			High independence		Low independence			High independence		Low independence		
	n	%	n	%	*P* value	n	%	n	%	*P* value	n	%	n	%	*P* value
**Protein foods**															
**Meat dishes**															
Every day	72	67.3	35	32.7	0.096	49	70.0	21	30.0	0.158	23	70.0	14	30.0	0.358
5–6 days a week	151	69.6	66	30.4		101	71.1	41	28.9		50	71.1	25	28.9	
3–4 days a week	606	72.7	227	27.3		436	74.8	147	25.2		170	74.8	80	25.2	
1–2 days a week	693	67.3	336	32.7		495	69.8	214	30.2		198	69.8	122	30.2	
Never	43	67.2	21	32.8		27	65.9	14	34.1		16	65.9	7	34.1	
**Soy products**															
Every day	642	74.1	224	25.9	< 0.001	426	77.2	126	22.8	< 0.001	216	67.9	98	32.1	0.002
5–6 days a week	261	73.7	93	26.3		185	75.8	59	24.2		76	64.9	34	35.1	
3–4 days a week	438	67.5	211	32.5		328	67.9	155	32.1		110	14.3	56	85.7	
1–2 days a week	218	59.7	147	40.3		168	64.9	91	35.1		50	73.8	56	26.2	
Never	6	37.5	10	62.5		1	14.3	6	85.7		5	75.1	4	24.9	
**Eggs/egg dishes**															
Every day	342	70.5	143	29.5	0.044	211	73.8	75	26.2	0.039	131	68.9	68	31.1	0.272
5–6 days a week	226	73.1	83	26.9		166	75.1	55	24.9		60	61.3	28	38.7	
3–4 days a week	553	70.4	233	29.6		398	72.2	153	27.8		155	75.2	80	24.8	
1–2 days a week	417	66.7	208	33.3		314	68.9	142	31.1		103	62.8	66	37.2	
Never	27	60.0	18	40.0		19	61.3	12	38.7		8	69.2	6	30.8	
**Bluefish**															
Every day	80	80.0	20	20.0	< 0.001	54	84.4	10	15.6	< 0.001	26	73.6	10	26.4	0.001
5–6 days a week	192	73.8	68	26.2		120	73.6	43	26.4		72	75.1	25	24.9	
3–4 days a week	605	72.5	230	27.5		428	75.1	142	24.9		177	67.3	88	32.7	
1–2 days a week	647	65.4	342	34.6		477	67.3	232	32.7		170	74.4	110	25.6	
Never	41	62.1	25	37.9		29	74.4	10	25.6		12	77.2	15	22.8	
**Other foods**															
**Vegetable dishes**															
Every day	1138	72.6	429	27.4	< 0.001	791	75.2	261	24.8	< 0.001	347	74.9	168	25.1	0.006
5–6 days a week	253	64.1	142	35.9		182	62.8	108	37.2		71	74.1	34	25.9	
3–4 days a week	140	61.4	88	38.6		110	69.2	49	30.8		30	65.6	39	34.4	
1–2 days a week	34	57.6	25	42.4		25	56.8	19	43.2		9	62.7	6	37.3	
Never	0	0.0	1	####		0	0.0	0	0.0		0	66.7	1	33.3	
**Fluits**															
Every day	932	72.2	359	27.8	< 0.001	637	74.9	214	25.1	< 0.001	295	75.8	145	24.2	0.070
5–6 days a week	243	71.1	99	28.9		180	74.1	63	25.9		63	73.2	36	26.8	
3–4 days a week	247	64.7	135	35.3		177	65.6	93	34.4		70	63.5	42	36.5	
1–2 days a week	133	60.7	86	39.3		106	62.7	63	37.3		27	56.9	23	43.1	
Never	10	62.5	6	37.5		8	66.7	4	33.3		2	56.9	2	43.1	
**Dairy products**															
Every day	950	69.9	409	30.1	0.124	653	73.2	239	26.8	0.014	297	75.8	170	24.2	0.685
5–6 days a week	199	75.1	66	24.9		141	75.8	45	24.2		58	73.2	21	26.8	
3–4 days a week	207	73.1	76	26.9		156	73.2	57	26.8		51	63.5	19	36.5	
1–2 days a week	169	62.6	101	37.4		129	63.5	74	36.5		40	56.9	27	43.1	
Never	40	54.8	33	45.2		29	56.9	22	43.1		11	56.9	11	43.1	

### Dietary patterns

PCA identified three components that explained 59.4% of the total variance (Table 4). The first component was characterized by dietary variety that showed that all food groups had a strong relationship with a high frequency of eating throughout the week. It was described as “dietary diversity.” The second pattern was a negative relationship for vegetable, fruit, and dairy products, which was called “low fruit, vegetable, and dairy product frequency.” The third pattern had a negative relationship with soy products and bluefish and a strong positive relationship with a high frequency of meat dishes during the week (principal component loading: 0.660); it was called “high meat frequency.”

**Table 4:**
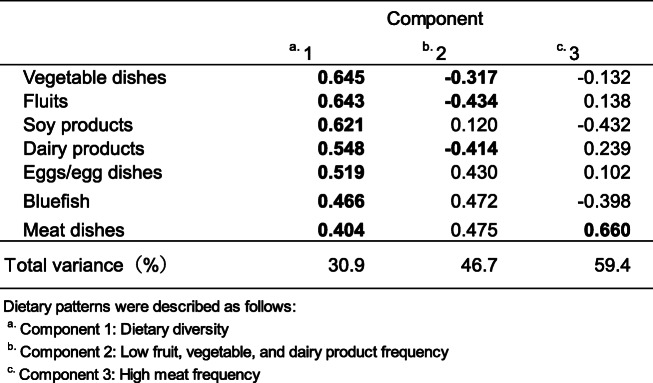
Principal component analysis for dietary patterns

### Comprehensive analysis for having low independence 3 years from baseline

At first, we tested all variables to determine whether there was an effect-modification (interaction) due to the baseline age stage or sex, with the IADL score after 3 years as the dependent variable. Baseline indicators that significantly interacted with the baseline age groups were frequency of soy products (F = 2.61, *P* = 0.034), vegetarian food (F = 4.66, *P* = 0.003), financial satisfaction (F = 4.66, *P* = 0.006), smoking Habits (F = 4.47, *P* = 0.012), and subjective health (F = 5.41, *P* = 0.001). We confirmed that gender interactions were not significantly associated with any variables.

Next, we conducted a multiple logistic regression analysis for having low independence 3 years after baseline. It was analyzed by age stage and sex separately using indices showing significant relationships with independence 3 years later, which showed no correlation coefficient of ≥0.5 between the indices.

First, we conducted the unadjusted single logistic regression analysis for being low independent 3 years after baseline with each individual dietary pattern derived from the PCA (Table 5). In earlier-stage older people, dietary diversity was associated with a significant lower risk of decline in independence 3 years after baseline regardless of sex (men: OR = 0.82, 95% CI: 0.72–0.94, women: OR = 0.75, 95% CI: 0.60–0.94). While high meat frequency was associated with a significant higher risk of decline in independence (men: OR = 1.16, 95% CI: 1.01–1.35, women: OR = 1.22, 95% CI: 1.00–1.48). On the other hand, in later-stage older people, a significantly lower risk of decline in independence for dietary diversity was shown only in men (OR = 0.75, 95% CI: 0.62–0.91), and the significant-higher risk of decline in independence for high meat frequency was observed only in women (OR = 1.64, 95% CI: 1.24–2.17).

**Table 5:**
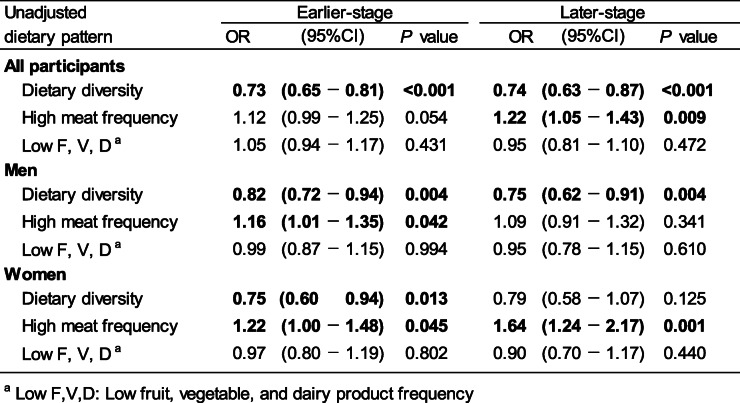
Relationship between each individual dietary pattern and having low independence three years after baseline

Multiple adjusted logistic regression analysis models compared all participants with age stage groups were then examined (Table 6). The indices that showed association with a significantly lower risk of decline in independence 3 years later for both age stages were the IADL score at baseline (earlier-stage older people: OR = 0.26, 95% CI: 0.21–0.32; later-stage older people: OR = 0.20, 95% CI: 0.14–0.27). The analysis by age group revealed that women were associated with a significantly lower risk of decline in independence 3 years later than men (women in earlier-stage: OR = 0.54, 95% CI: 0.38–0.75, women in the later-stage: OR = 0.60, 95% CI: 0.37–0.96).

**Table 6:** Multiple logistic regression analysis for having low independence 3 years after baseline by age stage

Baseline characteristics	All participants					Earlier-stage					Later-stage				
	(n = 2250)					(n = 1545)					(n = 705)				
	OR	(95%CI)			*P* value	OR	(95%CI)			*P* value	OR	(95%CI)			*P* value
**IADL score of baseline**	**0.24**	**(0.20**	**–**	**0.29)**	**< 0.001**	**0.26**	**(0.21**	**–**	**0.32)**	**< 0.001**	**0.20**	**(0.14**	**–**	**0.27)**	**< 0.001**
**Dietary pattern**															
Dietary diversity	**0.89**	**(0.80**	**–**	**0.99)**	**0.049**	0.89	(0.77	–	1.01)	0.078	0.92	(0.75	–	1.12)	0.406
Low fruit, vegetable, and dairy product frequency	1.02	(0.92	–	1.13)	0.723	1.02	(0.90	–	1.17)	0.728	1.01	(0.84	–	1.22)	0.922
High meat frequency	**1.18**	**(1.07**	**–**	**1.32)**	**0.002**	**1.19**	**(1.04**	**–**	**1.36)**	**0.010**	1.18	(0.98	–	1.41)	0.079
**Economic satisfaction**	1.04	(0.89	–	1.21)	0.620	1.05	(0.87	–	1.26)	0.624	0.98	(0.74	–	1.29)	0.869
**Salaried employee** (ref: No)	0.88	(0.67	–	1.15)	0.349	0.91	(0.67	–	1.25)	0.570	0.83	(0.49	–	1.41)	0.489
**No hospitalization over the past year** (ref: Yes)	0.81	(0.58	–	1.12)	0.194	0.80	(0.53	–	1.21)	0.287	0.85	(0.50	–	1.44)	0.541
**Subjective health**	0.90	(0.72	–	1.12)	0.353	0.98	(0.75	–	1.28)	0.868	0.73	(0.49	–	1.09)	0.128
**Life satisfaction**	1.03	(0.81	–	1.31)	0.818	1.09	(0.80	–	1.47)	0.598	0.95	(0.63	–	1.44)	0.804
**BMI categories** (ref: BMI < 18.5)															
18.5 < BMI < 25.0	**0.51**	**(0.31**	**–**	**0.84)**	**0.008**	**0.50**	**(0.25**	**–**	**0.99)**	**0.049**	0.55	(0.27	–	1.13)	0.101
25.0 < BMI < 30.0	0.64	(0.37	–	1.10)	0.103	0.65	(0.31	–	1.36)	0.255	0.61	(0.26	–	1.40)	0.244
30.0 < BMI	0.67	(0.24	–	1.86)	0.446	1.30	(0.36	–	4.65)	0.686	0.11	(0.01	–	1.10)	0.060
**Exercise frequency, enjoyment & fulfillment (E&F)** (ref:< 2 times/week)															
> 3 times/week, E&F	0.84	(0.62	–	1.13)	0.238	1.06	(0.72	–	1.54)	0.774	**0.54**	**(0.33**	**–**	**0.91)**	**0.020**
> 3 times/week	0.92	(0.57	–	1.48)	0.715	1.05	(0.56	–	1.95)	0.888	0.74	(0.34	–	1.61)	0.449
< 2 times/week, E&F	0.81	(0.58	–	1.11)	0.191	0.90	(0.60	–	1.35)	0.609	0.64	(0.37	–	1.12)	0.120
**No fall fractures in the past year** (ref: Yes)	**0.62**	**(0.45**	**–**	**0.84)**	**0.002**	**0.61**	**(0.41**	**–**	**0.92)**	**0.017**	0.61	(0.37	–	1.02)	0.057
**No cigarette smoking status**	0.97	(0.78	–	1.20)	0.762	0.85	(0.66	–	1.09)	0.197	1.34	(0.88	–	2.03)	0.168
**Community and volunteer activity engagement**(ref: Sometimes or No)	**0.66**	**(0.53**	**–**	**0.82)**	**< 0.001**	**0.62**	**(0.47**	**–**	**0.81)**	**< 0.001**	0.77	(0.53	–	1.12)	0.167
**Sex** (ref: Men)	**0.56**	**(0.43**	**–**	**0.73)**	**< 0.001**	**0.54**	**(0.38**	**–**	**0.75)**	**< 0.001**	**0.60**	**(0.37**	**–**	**0.96)**	**0.031**
**Age** (+ 1 years old)	**1.03**	**(1.01**	**–**	**1.05)**	**0.004**	1.00	(0.95	–	1.05)	0.947	1.02	(0.96	–	1.08)	0.582

The dietary diversity was associated with a lower risk of decline in independence 3 years later in both age stages (Table 5); however, these effects were attenuated after adjusting for other dietary patterns or IADL-related factors and became insignificant (Table 6). Conversely, high meat frequency was associated with a significantly higher risk of decline in independence in earlier-stage individuals after adjusting for IADL related factors (OR = 1.19, 95%CI: 1.04–1.36).

The earlier-stage older people showed an association with a significantly lower risk of decline in independence 3 years later for 18.5 ≤ BMI < 25 (OR = 0.50, 95% CI: 0.25–0.99, reference group: BMI < 18.5). A BMI ≥ 30.0 tended to be a higher risk for decline in independence in earlier-stage older people (OR = 1.30, 95% CI: 0.36–4.65), albeit a lower risk in all participants (OR = 0.67, 95% CI: 0.24–1.86) and in later-stage individuals (OR = 0.11, 95% CI: 0.01–1.10).

In the earlier-stage older people, no fall fractures in the previous year (OR = 0.61, 95% CI: 0.41–0.92) and community and volunteer activity engagement (OR = 0.62, 95% CI: 0.47–0.81) were associated with a significantly lower risk of decline in independence 3 years later. Meanwhile, only the later-stage older people showed an association with a significantly lower risk for ≥3 times/week with E&F (OR = 0.54, 95% CI: 0.33–0.91, reference group: ≤2 times/week).

Subsequently, the analysis by age stage and sex was conducted (Table 7). The high meat frequency in the later-stage older women was associated with the highest risk of decline in independence 3 years later for all results, regardless of sex or age (OR = 1.67, 95% CI: 1.18–2.35). The results revealed that earlier-stage older men showed an association with a higher risk of decline in independence for a BMI ≥ 30 (OR = 2.24, 95% CI: 0.25–19.9), albeit not significant, and with a significantly lower risk of decline in independence for community and volunteer activity engagement (E&F) (OR = 0.49, 95% CI: 0.35–0.68). The later-stage older men showed an association with a lower risk of decline in independence for ≥3 times/week with E&F (OR = 0.46, 95% CI: 0.24–0.88, reference group: ≤2 times/week). The results of earlier-stage older women showed an association with a lower risk of decline in independence for no fall fractures in the previous year (OR = 0.42, 95% CI: 0.24–0.75).

**Table 7:** Multiple logistic regression analysis for having low independence 3 years after baseline by age stage and sex

Men											Women									
Baseline characteristics	Earlier-stage					Later-stage					Earlier-stage					Later-stage				
	(*n* = 863)					(*n* = 431)					(*n* = 682)					(*n* = 274)				
	OR	(95%CI)			*P* value	OR	(95%CI)			*P* value	OR	(95%CI)			*P* value	OR	(95%CI)			*P* value
**IADL score of baseline**	**0.27**	**0.22**	**–**	**0.34)**	**< 0.001**	**0.21**	**(0.14**	**–**	**0.31)**	**< 0.001**	**0.20**	**(0.13**	**–**	**0.29)**	**< 0.001**	**0.13**	**(0.06**	**–**	**0.28)**	**< 0.001**
**Dietary pattern**																				
Dietary diversity	0.88	(0.75	–	1.04)	0.133	0.90	(0.71	–	1.14)	0.387	0.88	(0.67	–	1.15)	0.344	0.91	(0.60	–	1.36)	0.641
Low fruit, vegetable, and dairy product frequency	1.00	(0.85	–	1.18)	0.999	1.05	(0.83	–	1.32)	0.700	1.08	(0.86	–	1.37)	0.508	1.01	(0.71	–	1.42)	0.977
High meat frequency	**1.23**	**(1.04**	**–**	**1.46)**	**0.016**	1.02	(0.82	–	1.27)	0.866	1.11	(0.89	–	1.39)	0.351	**1.67**	**(1.18**	**–**	**2.35)**	**0.004**
**Economic satisfaction**	1.06	(0.83	–	1.36)	0.626	1.01	(0.71	–	1.43)	0.966	1.01	(0.75	–	1.36)	0.936	0.87	(0.54	–	1.41)	0.575
**Salaried employee** (ref: No)	1.00	(0.68	–	1.46)	0.992	0.76	(0.39	–	1.48)	0.418	0.71	(0.39	–	1.29)	0.256	1.22	(0.49	–	3.09)	0.669
**No hospitalization over the past year** (ref: Yes)	0.76	(0.46	–	1.23)	0.261	0.70	(0.36	–	1.38)	0.304	1.03	(0.45	–	2.36)	0.943	1.22	(0.46	–	3.23)	0.683
**Subjective health**	0.86	(0.60	–	1.23)	0.408	0.78	(0.47	–	1.31)	0.353	1.08	(0.69	–	1.67)	0.747	0.61	(0.31	–	1.20)	0.151
**Life satisfaction**	1.26	(0.84	–	1.88)	0.259	0.96	(0.56	–	1.66)	0.886	0.90	(0.55	–	1.47)	0.663	1.05	(0.52	–	2.11)	0.901
18.5 < BMI < 25.0	0.33	(0.09	–	1.28)	0.110	0.66	(0.22	–	1.99)	0.461	0.58	(0.25	–	1.37)	0.214	0.43	(0.15	–	1.20)	0.107
25.0 < BMI < 30.0	0.47	(0.12	–	1.87)	0.285	0.69	(0.21	–	2.30)	0.547	0.61	(0.22	–	1.69)	0.338	0.60	(0.16	–	2.26)	0.451
30.0 < BMI	2.24	(0.25	–	19.9)	0.469	–				0.999	0.70	(0.11	–	4.70)	0.717	0.17	(0.01	–	2.75)	0.213
> 3 times/week, E&F	0.96	(0.58	–	1.58)	0.865	**0.46**	**(0.24**	**–**	**0.88)**	**0.018**	1.29	(0.71	–	2.37)	0.404	0.79	(0.32	–	1.98)	0.619
> 3 times/week	1.24	(0.57	–	2.70)	0.597	0.69	(0.25	–	1.91)	0.473	0.87	(0.29	–	2.64)	0.804	0.91	(0.24	–	3.41)	0.888
< 2 times/week, E&F	0.94	(0.55	–	1.60)	0.807	0.54	(0.26	–	1.13)	0.102	0.79	(0.40	–	1.56)	0.497	0.99	(0.39	–	2.51)	0.990
**No fall fractures in the past year** (ref: Yes)	0.84	(0.47	–	1.51)	0.566	0.77	(0.38	–	1.55)	0.464	**0.42**	**(0.24**	**–**	**0.75)**	**0.003**	0.55	(0.25	–	1.23)	0.147
**No cigarette smoking status**	0.82	(0.62	–	1.08)	0.158	1.33	(0.87	–	2.03)	0.189	1.02	(0.54	–	1.93)	0.948	1.62	(0.22	–	11.9)	0.636
**Community and volunteer activity engagement**(ref: Sometimes or No)	**0.49**	**(0.35**	**–**	**0.68)**	**< 0.001**	0.87	(0.54	–	1.38)	0.545	0.92	(0.58	–	1.46)	0.723	0.62	(0.31	–	1.23)	0.169
**Sex** (ref: Men)	–					–					–					–				
**Age** (+ 1 years old)	1.00	(0.95	–	1.07)	0.912	1.01	(0.94	–	1.08)	0.856	1.00	(0.93	–	1.09)	0.923	1.04	(0.94	–	1.15)	0.458
OR:odds ratio, CI:confidence interval, ref.:refference																				

### Multiple-group path analysis for testing the difference among age stage and sex

To test the difference among groups of age stage and sex, we conducted Multiple-Group Path Analysis (i.e., analyzing multiple groups simultaneously). A Path analysis of SEM was used to examine the direct effect of 15 indicators (those in Tables 6 and 7) of baseline characteristics on low independence 3 years later. Each effect was labeled in order. For example, in the age stage and sex group, the direct effect of earlier-stage men’s IADL baseline score was labeled as par_1, and the last label was par_60 for the effect of later-stages women’s baseline age. S.E. and CR to the reference group are shown in Table 8. Only the results of the baseline indicators, with significant odds ratios in Tables 6 and 7, are listed. Negative S.E. indicated that older people were more likely to be at lower risk of low independence after 3 years.

**Table 8:**
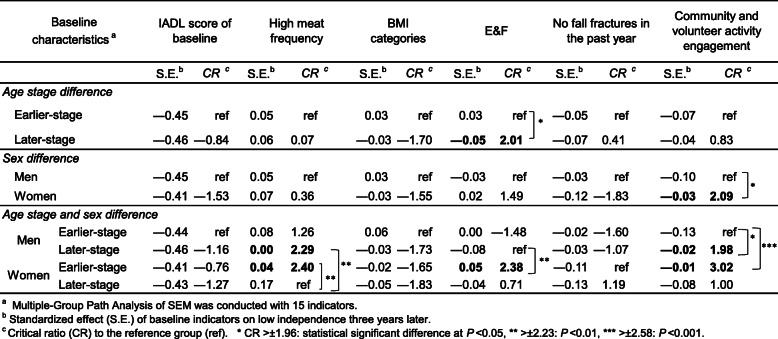
Standardized effect by Multiple-Group Analysis and its difference between reference group.

The results that had significant associations are as follows. For the age stage difference, there was a significant difference in the magnitude of the effects of E&F on low independence 3 years later in earlier-stage and later-stage individuals. The effect of community and volunteer activity engagement of men was significantly larger than women. For the age stage and sex difference, the effect of high meat frequency pattern among later-stage older women was significantly larger than among later-stage older men and earlier-stage older women. The result of E&F of later-stage older men was significantly different from earlier-stage older women and community and volunteer activity engagement of earlier-stage older men was significantly larger than later-stage older men and earlier-stage older women.

## Discussion

In providing evidence for building a comprehensive support strategy for preventing a decline in independence, this study conducted a comprehensive analysis of IADL-related multidimensional factors, especially several dietary patterns, and a decline in independence after 3 years, by age stage of two groups and sex separately. In this study, the outcome of the analysis was set for low independence, defined as the participants without a full score for IADL. Only the later-stage older peoples’ proportion of low independence after 3 years was significantly higher than those at baseline. The results suggest that later-stage older people in particular need more urgent health care support than earlier-stage people.

### Relationship between dietary patterns and low independence 3 years later

Our study investigated three dietary patterns in the participants: dietary diversity, low fruit, vegetable, dairy product frequency, and high meat frequency. Usually, dietary patterns are examined separately with the outcome factor; however, there is a possibility of obtaining valuable results for accumulating helpful evidence for health care strategies [7]. In our multivariate analysis, three kinds of dietary patterns were used simultaneously, and we investigated the impact of every pattern for low independence 3 years later. Because principal components derived from the PCA will be uncorrelated, they are a good fit for use as independent variables for logistic regression analysis [23].

After adjusting for other dietary patterns or IADL-related factors, the relationship between dietary diversity and decline in independence after 3 years became insignificant in both age stages. Simultaneously, high meat frequency was associated with a higher risk of decline in independence. Notably, later-stage older women showed the highest association with the risk of IADL disability. Consistent with results from other dietary pattern studies of older people [13], this study suggests that unhealthy dietary patterns, such as high meat intake, might impact the decline in independence on later-stage older people than rich dietary patterns of dietary diversity.

Diets with a high intake of animal protein, such as meats rich in saturated fatty acids, and a low intake of bluefish, rich in n-3 fatty acids, and soy-based foods with vegetable protein may promote a decline in independence. Meat may play a key role in the relationship between inflammation and low independence in older people. The Dietary Inflammatory Index (DII) [26] was created by Shivappa et al. to score 45 types of foods and nutrients based on their inflammatory properties, according to a review of 1943 research papers. DII scores have been validated using inflammatory markers, such as blood C-reactive protein (CRP), to indicate chronic inflammation [26]. The DII score of saturated fatty acids, which are abundant in meat, was 0.373. It was the highest possible score and was evaluated as the highest pro-inflammatory nutrient.

Conversely, the n-3 fatty acid content in bluefish was − 0.436. Using the National Health and Nutrition Survey Data of 2572 Japanese adults, a positive association was found between DII scores and CRP. The higher the DII score, the higher the likelihood of observing high meat intake in Japanese adults [27]. Many studies have reported that a high-pro-inflammatory diet with high DII scores in older people is associated with disability or death [28].

In recent years, high protein intake by older people has been recommended as a support for the decline in independence prevention. The recommendation of eating moderate amounts of meat and other food varieties is necessary, especially for later-stage older people, and should be included in nutrition education.

### Comprehensive analysis of low independence after 3 years

We carried out a comprehensive analysis by all participants and by age stages. By comparing those results, we clarified each age group’s characteristics that are not revealed by all participants’ results. Furthermore, those differences among age stage or sex were confirmed statistically.

In this study, an appropriate BMI was associated with preventing a decline in earlier-stage older men’s independence. Earlier-stage older people were between 65 and 75 years of age, suggesting the necessity of continuing measures, such as weight management, to prevent chronic lifestyle diseases and aggravation. The high frequency of saturated fatty acid-rich meat in the earlier-stage may be associated with the risk of decline in independence mediated by obesity-related lifestyle-related diseases. Simultaneously, in the later-stage, it was expected that decline could be more strongly affected by inflammation risk.

Furthermore, social participation, such as community and volunteer activity engagement, could prevent a disability in earlier-stage older people, especially for men. The use of resources other than specialists has also been reported [29]. For example, utilizing earlier-stage older people as leaders in volunteer activities in an independence decline prevention program for the latter group may present a model of support in the future.

Our results for the later-stage older people suggest that exercise frequency and E&F may be priority support items for preventing a decline in independence after 3 years. A previous study of people aged 85 years and older reported that, even if the effective aerobic exercise consisting of 3 times per week was effective for older people’s physical function after 6 months, it did not have a significant effect on their mental health [30]. The vulnerability of mental and psychological factors in older people is a multifaceted problem associated with declining independence. This study suggests that mental and psychological factors are essential needs, especially for later-stage older people.

Social participation might be one of the target factors that lead to the realization of successful aging to meet older people’s needs for mental and physical health [14]. Our study also showed that “community and volunteer activity engagement” appeared to prevent low independence after 3 years in all age stages for both sexes. Because social support and social cohesion mediate the relationship between social participation and older people’s health [14], older people who have more chances for social participation are more likely to receive support from community care, resulting in more physical activities [31].

### Limitations

This study had several limitations. First, we evaluated the respondents’ independence using the validated TMIG-IC, which asked for subjective information on the participants’ ability to engage in IADL. Utilizing a numerical index as an outcome, such as healthy life expectancy, which has been calculated by a period of not needing nursing or support [32], would have provided effective evidence for making a strategy for health care support.

Next, dietary patterns were derived based on the frequency of food consumption. Although the BMI related to total intake was added to the multivariate-adjusted model analysis, it was not calculated based on a quantitative intake of multiple single food items. Furthermore, there are different approaches for assessing dietary patterns, namely, investigator-defined patterns methods, for example, “DASH” or “Mediterranean diet scores.” Future studies should examine these practical methods further.

## Conclusions

The present study was conducted for the community-dwelling independent older people in Japan, analyzing earlier and later-stage older people in both sexes. The results showed that the IADL disability in older people aged 75 and over was a rapid change and revealed that the characteristics associated with the risk of decline in independence 3 years later were statistically different among age stage and sex. It is necessary to implement community-based comprehensive supportive strategies to prevent disability in older people for long life globally, targeting age stages and sex separately.

## Data Availability

The data that support the findings of this study are available from the Tokyo Metropolitan University. However, restrictions apply to the data availability, used under the current study’s license, and are not publicly available. Nevertheless, data are available from the authors upon reasonable request and permission from the Tokyo Metropolitan University.

## Abbreviations

BMI: BODY mass index
CI: Confidence interval
CR: Critical ratio
DASH: Dietary approaches to stop hypertension
IADL: Instrumental activities of daily living
ICOPE: Integrated care for older people
n-3: Omega-3
n-6: Omega-6
OR: Odds ratio
SEM: Structural Equation Modeling
S.E.: Standardized estimates
TMIG-IC: Tokyo Metropolitan Institute of Gerontology Index of Competence
FSDSC: Foundation of Social Development for Senior Citizens
