# Correction to: Decline in independence after three years and its association with dietary patterns and IADL-related factors in community-dwelling older people: an analysis by age stage and sex

**DOI:** 10.1186/s12877-021-02384-7

**Published:** 2021-09-21

**Authors:** Sayuri Kodama, Tanji Hoshi, Sugako Kurimori

**Affiliations:** 1 Department of Food and Nutrition Science, Sagami Women’s Junior College, 2-1-1 Bunkyo, Minami-ku, Sagamihara-shi, Kanagawa-ken 252-0383 Japan; 2 Tokyo Metropolitan University, 1-1 Minami-Osawa, Hachioji-shi, Tokyo 192-0397 Japan; 3 Department of Nursing, Seitoku University, 550 Iwase, Matsudo-shi, Chiba-ken 271-8555 Japan

**Correction to: BMC Geriatr 21, 385 (2021)**

**https://doi.org/10.1186/s12877-021-02332-5**

Following publication of the original article [1], it was reported that the indentation in Tables 6 and 7 was incorrect.

The original article [1] has been corrected.
